# Supplementary material for: Prevalence and Risk Factors for ESBL/AmpC-E. coli in Pre-Weaned Dairy Calves on Dairy Farms in Germany
Source: Microorganisms. 2021 Oct 12;9(10):2135. doi: 10.3390/microorganisms9102135 (PMC8539614; doi:10.3390/microorganisms9102135)
Supplement: Supplementary file 1 [file microorganisms-09-02135-s001.zip › Supplement_1.pdf]

Supplement 1: Total number of calves within the farms aged 7-28 days at the time of sampling and the sample size calculated using an estimated prevalence of 10%, a confidence Level of 95%, and a desired precision of 5%.

farm	total number of calves on farm at date of sampling	sample size calculated	calves sampled
**1**	65	45	45
**2**	6	6	6
**3**	8	8	8
**4**	20	18	18
**5**	12	12	12
**6**	8	8	8
**7**	10	10	10
**8**	7	7	7
**9**	5	5	5
**10**	25	22	22
**11**	27	23	23
**12**	24	21	21
**13**	46	35	35
**14**	59	42	42
**15**	32	27	27
**16**	24	21	21
**17**	37	30	30
**18**	17	16	16
**19**	31	26	26
**20**	10	10	10
**21**	16	15	15
**22**	7	7	7
**23**	14	13	13
**24**	32	27	27
**25**	11	11	11
**26**	15	14	14
**27**	24	21	21
**28**	24	21	21
**29**	13	12	12
**30**	10	10	10
**31**	28	24	24
**32**	21	19	19
**33**	19	17	17
**34**	30	25	25
**35**	5	5	5
**36**	32	27	27
**37**	36	29	29
**38**	14	13	13

**39**	32	27	27
**40**	15	14	14
**41**	19	17	17
**42**	94	57	57
**43**	11	11	11
**44**	23	20	20
**45**	27	23	23
**46**	63	44	44
**47**	21	19	19
**48**	39	31	31
**49**	19	22	22
**50**	30	25	25
**51**	53	39	39
**52**	22	19	19
**53**	9	9	9
**54**	28	24	24
**55**	32	27	27
**56**	20	18	18
**57**	14	13	13
**58**	24	21	21
**59**	27	23	23
**60**	17	16	16
**61**	24	21	21
**62**	31	26	26
**63**	8	8	8
**64**	32	27	27
**65**	27	23	23
**66**	37	30	30
**67**	7	7	7
**68**	17	16	16
**69**	33	27	27
**70**	17	16	16
**71**	12	12	12
**72**	8	8	8
