# Supplementary material for: Prevalence and Risk Factors for ESBL/AmpC-E. coli in Pre-Weaned Dairy Calves on Dairy Farms in Germany
Source: Microorganisms. 2021 Oct 12;9(10):2135. doi: 10.3390/microorganisms9102135 (PMC8539614; doi:10.3390/microorganisms9102135)
Supplement: Supplementary file 1 [file microorganisms-09-02135-s001.zip › Supplement_2.pdf]

Supplement 2. Questionnaire of the study Prevalence and risk factors for the occurrence of ESBL *E. coli* in pre-weaned dairy calves and their dams on large dairy farms in Germany - a cross-sectional study

Data of the participants

General Data

○ conventional farming

○ organic farming (since ________________)

■Are the other livestock husbandries within a radius of 3 km?

○ yes, ____(species)

○ no

■Frequently buy-in of cattle?

○ yes ○ no

If yes, how often?

○ more than once a month

○ every 1-3 months

○ less than every 3 months

Information of the origin farm/s

○ only 1 origin farm

○ > 1 fixed origin farms

○ > 1 changing farms

■Outsourced heifer rearing?

○ yes ○ no

If yes, in

○ another location of the own farm

○ an external farm

○ heifers are raised at more than 1 location ______ (number)

○ there are “foreign“ animals at this location

■Which animals feeds are used for calves and cows:

○ in-house basic forage____

○ in-house substrates____

○ external basic forage____

○ external substrates____

○ bought in forage comes from a single farm

○ bought in forage comes from different farms

How often do the producers change?

○ > 1 x / month

○ every 1-3 months

○ less than every 3 months

■Fertilization of the fields that are used for the feed/bedding material production

○ in-house slurry

○ external slurry

■Does an in-house biogas reactor exist?

○ yes ○ no

If yes, do you use in-house material exclusively?

○ yes ○ no, ____

Are the fields that are used for feed production or bedding material fertilized with the outcome of the biogas plant, that were fed with foreign slurry/manure?

○ yes ○ no

How many foreign husbandries are involved?

○ 1-3

○ more than 3

How often do foreign husbandries change?

○ never

○ more than every 3 months

○ less than every 3 months

Which foreign husbandries are we talking about? (species)

○ ___________________

○ ___________________

○ ___________________

■Which people have contact with the livestock animals?

○ personnel

○ veterinarian

○ family members

○ visitors (e. g. providers, craftsman)

Do this people have contact to other animals?

○ yes, to____(species)

○ no

■Do you perform pest control?

○ yes, ____

○ no

Calving management

Housing: ○ single pen ○ group pen

○ straw bedding ○ ____________

Hygiene-management:

Cleaning frequency?

○ after each birth

○ more than once a week

○ less than once a week

How long do calves stay with their dams after calving?

○ <1h

○ 1-3h

○ >3h

Co-husbandry of calving and dry cows?

○ yes, in more than half of the calvings

○ yes, in less than half of the calvings

○ no

Co-husbandry of calving and sick cows?

○ yes, in more than half of the calvings

○ yes, in less than half of the calvings

○ no

<u>Calves up to 28d</u>

Housing:

○ single Igloo (____%) ○ single pen (____%)

○ group Igloo (____%) ○ group pen (____%)

■Change of the housing:

○ 2 weeks

○ no change

○______________

New housing:

○ group igloo (____%)

○ group pen (____%)

■Floor:

○ concrete floor

○ natural floor

■Bedding material and frequency:

○ straw

○ daily bedding

○ bedding if necessary

■Direct contact to other calves:

○ yes

○ no

■Number of calves per group:

○ calve-feedlot Ratio >1:1

○ calve-feedlot Ratio <1:1

■Direct contact to other animals

○ yes

○ no

○ ____ (species)

■All-in-all-out-principle

○ yes

○ no

If yes,

○ all calves

○ not for all calves (____%)

Feeding:

■<u>Colostrum feeding:</u>

○ immediately after birth

○ until 3 h after birth

○ later than 3 h

○ colostrum of the own dam (○ milked off ○ directly from the dam)

○ colostrum stored frozen and defrosted

○ colostrum stored in the refrigerator

○ colostrum is pasteurized

○ colostrum is acidified (with_______________)

○ the dams have been present on the farm for at least 3 weeks before birth

○ foreign colostrum is occasionally used

origin farm: ____

How often? ________________________ How many calves? _________________)

○ check of colostrum quality

How? ____________________________________

How much colostrum do calves receive? _________________________________

How?

○ drench ○ nursing bucket

<u>Further feeding management:</u>

■○ all calves ○ heifer calves

Age: from __________________ to ___________________

○ raw milk

○ waste milk (antibiotic residues)

○ waste milk (without antibiotic residues)

○ milk replacer

○ __ x per day, _______________(volume)

○ ad libitum

○ milk is pasteurized

○ milk is acidified

Age: From __________________ to ___________________

○ raw milk

○ waste milk (antibiotic residues)

○ waste milk (without antibiotic residues)

○ milk replacer

○ __ x per day, _______________(volume)

○ ad libitum

○ milk is pasteurized

○ milk is acidified

■○ male calves

Age: From __________________ to ___________________

○ raw milk

○ waste milk (antibiotic residues)

○ waste milk (without antibiotic residues)

○ milk replacer

○ __ x per day, _______________(volume)

○ ad libitum

○ milk is pasteurized

○ milk is acidified

Age: From __________________ to ___________________

○ raw milk

○ waste milk (antibiotic residues)

○ waste milk (without antibiotic residues)

○ milk replacer

○ __ x per day, ________________(volume)

○ ad libitum

○ milk is pasteurized

○ milk is acidified

■Feeding practice?

○ always at fixed times

○ irregularly

○ use of automatic feeder (○ freshly prepared, ○stored)

■How is the calve feeder cleaned and disinfected?

○ no cleaning and disinfection

○ with water only

○ with cleaning agent

○ with cleaning and disinfectant

How often is the calf feeder cleaned and disinfected?

○ after each meal

○ 1 x / day

○ < 1 x / day

■Do calves receive supplementary feed?

○ yes, roughage from day ________________________

○ yes, concentrate from day ________________________

○ no

What is used to prepare the milk replacer?

○ tap water

○ well water

■Do the calves have free water access?

○ yes ○ no

○ tap water

○ well water

■Which people take care of the calves?

○ number____

○ trained ○ untrained

○ changing ○ constant

Use of protective clothing?

○ yes

○ no

■Use of preventive treatment for calves?

○ yes, ____
____
____

○ no

How do you assess calf health? What are the biggest problems?

What do you do about it?

Use of treatment schedules for claves?

○ yes

○ no

Cows

Housing systems:

○ cubicle housing system (____%)

○ deep-bedded straw yard housing system (____%)

○ flat floors (____%) ○ slatted floors (____%)

○ high cubicle beds (____%) ○ low cubicle beds (____%)

○ straw (____%)

○ sawdust (____%)

○ ____________________________________

○ lime (____%)

○ pasture (____%)

Dry-off-management:

Cows are dried with antibiotic treatment________________________________(name)

Use of teat sealant?

○ yes

○ no

Cows are dried ____________ days/weeks bevore birth date.

Use of maternal vaccinations?

○ yes, Rota/Corona/E.coli

○ yes, ____________________________

○ no

Milking routine:

Udder preparation:

○ dry cleaning

○ wet cleaning __________________

○ pre-dip

○ milked by hand

○ stimulated per brush __________

Use of post-milking treatment

○ yes, with ______________________

○ dip

○ spray

Use of milking cluster disinfection

○ yes, with ______________________

○ no

Use of separate milking parlor for sick cows?

○ yes

○ no

How is the milking order?

○ cows with waste milk at the end

○ cows with high somatic cell count at the end

○ mixed order

Health management:

Use of preventive treatment for cows?

○ yes, ____ (____%)

○ no

Use of treatment schedules for cows?

○ yes

○ no

Is every clinical mastitis treated with antibiotics?

○ yes, with____ (____%)

○ no

Other treatment schedules:

If a treatment doesn´t improve the health of the animal, when do you change the antibiotic treatment?

○ never (____%)

○ after 1 day (____%)

○ after 2 days (____%)

○ after 3 days (____%)

○ only after completion of the treatment (____%)

Use of alternative treatment methods, e. g. homeopathy?

○ yes, ____ (____%)

○ no

Housing of the cows producing waste milk?

○ in the regular group (____%)

○ separated with other waste milk cows (____%)

○ separated with other cows, that were treated with antibiotics (____%)

Cleaning and disinfection management:

How does the cleaning and disinfection proceed?

Calving pen	Calf housing	Milking parlor
Cleaner<br><br>○ we do not use any<br>cleaning agent<br><br>Exposure time<br>○ shorter than the manufacturer's instructions<br>○ according to the manufacturer's instructions	Cleaner<br><br>○ we do not use any<br>cleaning agent<br><br>Exposure time<br>○ shorter than the manufacturer's instructions<br>○ according to the manufacturer's instructions	Cleaner<br><br>○ we do not use any<br>cleaning agent<br><br>Exposure time<br>○ shorter than the manufacturer's instructions<br>○ according to the manufacturer's instructions
Is there complete dry-through?	Is there complete dry-through?	Is there complete dry-through?

○ yes<br>○ no	○ yes<br>○ no	○ yes<br>○ no
Disinfectant<br><br>○ we do not use a disinfectant<br><br>Exposure time<br>○ shorter than the manufacturer's instructions<br>○ according to the manufacturer's instructions	Disinfectant<br><br>○ we do not use a disinfectant<br><br>Exposure time<br>○ shorter than the manufacturer's instructions<br>○ according to the manufacturer's instructions	Disinfectant<br><br>○ we do not use a disinfectant<br><br>Exposure time<br>○ shorter than the manufacturer's instructions<br>○ according to the manufacturer's instructions
Use of cleaning and disinfection plan<br><br>○ yes<br>○ no	Use of cleaning and disinfection plan<br><br>○ yes<br>○ no	Use of cleaning and disinfection plan<br><br>○ yes<br>○ no
Cleaning agents?<br><br>○ Surfactants<br>○ Acids<br>○ Bases<br>○ Bleaching agents<br>○ Enzymes	Cleaning agents?<br><br>○ Surfactants<br>○ Acids<br>○ Bases<br>○ Bleaching agents<br>○ Enzymes	Cleaning agents?<br><br>○ Surfactants<br>○ Acids<br>○ Bases<br>○ Bleaching agents<br>○ Enzymes
Disinfectants?<br><br>○ Peracetic acid<br>○ Hydrogen peroxide<br>○ Chlorine<br>○ Iodine<br>○ Aldehydes<br>○ Alcohols	Disinfectants?<br><br>○ Peracetic acid<br>○ Hydrogen peroxide<br>○ Chlorine<br>○ Iodine<br>○ Aldehydes<br>○ Alcohols	Disinfectants?<br><br>○ Peracetic acid<br>○ Hydrogen peroxide<br>○ Chlorine<br>○ Iodine<br>○ Aldehydes<br>○ Alcohols

o Phenols<br><br>o Nitrogen compounds	o Phenols<br><br>o Nitrogen compounds	o Phenols<br><br>o Nitrogen compounds

Assessment of the herd manager

What is the biggest health problem of the herd?

____

____

How many animals do you treat with antibiotics per day on average?

o <2 per 100 animals treated

o 2-5 per 100 animals treated

o 5-10 per 100 animals treated

o >10 per 100 animals treated

Which antimicrobial ingredients were used in the farm in the last 6 months?

o β-lactam antibiotics (o penicillins o cephalosporins)

o tetracyclines

o aminoglycosides

o Polypeptides

o Macrolides

o Fenicols

o Folic acid antagonists

o Chinolons
