# Supplementary material for: Prevalence and Risk Factors for ESBL/AmpC-E. coli in Pre-Weaned Dairy Calves on Dairy Farms in Germany
Source: Microorganisms. 2021 Oct 12;9(10):2135. doi: 10.3390/microorganisms9102135 (PMC8539614; doi:10.3390/microorganisms9102135)
Supplement: Supplementary file 1 [file microorganisms-09-02135-s001.zip › Supplement_3.pdf]

Supplement 3: Responses of the participating farms (total and percentage) to the Questionnaire of the study Prevalence and risk factors for the occurrence of ESBL *E. coli* in pre-weaned dairy calves and their dams on large dairy farms in Germany—a cross-sectional study

		Applicable		not applicable		no specification	
		total	%	total	%	total	%
**1**	Conventional farming	71	98,6	1	1,4	0	0
**2**	Organic farming	1	1,4	71	98,6	0	0
**3**	Cattle husbandry in 3 km radius	44	61,1	26	36,1	2	2,8
**4**	Horse husbandry in 3 km radius	21	29,2	49	68,1	2	2,8
**5**	Pig husbandry in 3 km radius	28	38,9	42	58,3	2	2,8
**6**	Goat/sheep husbandry in 3 km radius	7	9,7	63	87,5	2	2,8
**7**	Poultry husbandry in 3 km radius	9	12,5	61	84,7	2	2,8
**8**	Regular buy-in of livestock	22	30,6	50	69,4	0	0
**8a**	Buy-in > 1 x per month	2	2,8	70	97,2	0	0
**8b**	Buy-in every 1-3 months	11	15,3	61	84,7	0	0
**8c**	Buy-in less than every 3 months	3	4,2	69	95,8	0	0
**8d**	From one fixed origin farm	10	13,9	62	86,1	0	0
**8e**	Several, fixed origin farms	6	8,3	66	91,7	0	0
**8f**	Several, changing origin farms	6	8,3	66	91,7	0	0
**9**	Outsourced heifer rearing	51	70,8	21	29,2	0	0
**9a**	In another location of the own farm	29	40,3	43	59,7	0	0
**9b**	In another farm	23	31,9	49	68,1	0	0
**9c**	In several other locations	10	13,9	62	86,1	0	0
**9d**	With other foreign animals	9	12,5	63	87,5	0	0
**10**	In-house production of basic feed	40	55,6	32	44,4	0	0
**11**	Purchase of basic feed	29	40,3	43	59,7	0	0
**11a**	From one producer	9	12,5	63	87,5	0	0
**11b**	From several producers	61	84,7	11	15,3	0	0
**11c**	Change more than once a month	3	4,2	69	95,8	0	0
**11d**	Change every 1- 3 months	3	4,2	69	95,8	0	0
**11e**	Change less than every 3 months	65	90,3	7	9,7	0	0
**12**	In-house and external manure as fertilizer	14	19,4	58	80,6	0	0
**13**	Only external manure as fertilizer	3	4,2	69	95,8	0	0
**14**	In-house biogas plant	48	66,7	24	33,3	0	0
**14a**	Use of in-house material	34	47,2	38	52,8	0	0
**14b**	Use of external material	12	16,7	60	83,3	0	0
**14c**	Less than 3 suppliers	9	12,5	63	87,5	0	0
**14d**	More than 3 suppliers	1	1,4	71	98,6	0	0
**14e**	Fixed suppliers	8	11,1	64	88,9	0	0
**14f**	Change less than every 3 months	3	4,2	69	95,8	0	0
**14g**	Manure from cattle farms	3	4,2	69	95,8	0	0
**14h**	Manure from pig farms	4	5,6	68	94,4	0	0
**14i**	Manure from horse farms	3	4,2	69	95,8	0	0

**14j**	Manure from poultry farms	1	1,4	71	98,6	0	0
**15**	Changing persons have contact to the dairy herd	72	100	0	0	0	0
**15a**	Persons have further animal contact	72	100	0	0	0	0
**15b**	Use of in-house protective clothing	1	1,4	71	98,6	0	0
**16**	Pest control	67	93,1	5	6,9	0	0
**16a**	Rodents	33	45,8	39	54,2	0	0
**16b**	Flies	2	2,8	70	97,2	0	0
**16c**	Rodents and flies	32	44,4	40	55,6	0	0
**17**	Cubicle housing system > 50%	72	100	0	0	0	0
**18**	Flat floors > 50%	38	52,8	34	47,2	0	0
**19**	Slatted floors > 50%	34	47,2	38	52,8	0	0
**20**	High cubicle beds > 50%	31	43,1	41	56,9	0	0
**21**	Low cubicle beds > 50%	37	51,4	35	48,6	0	0
**22**	50% high/ 50% low cubicle beds	2	2,8	70	97,2	0	0
**23**	Straw	37	51,4	35	48,6	0	0
**24**	Sawdust	7	9,7	65	90,3	0	0
**25**	Other bedding material	16	22,2	56	77,8	0	0
**26**	Use of lime	51	70,8	21	29,2	0	0
**27**	Dry teat cleaning	15	20,8	57	79,2	0	0
**28**	Wet teat cleaning	30	41,7	42	58,3	0	0
**29**	Direct milking	3	4,2	69	95,8	0	0
**30**	Post-teat treatment	24	33,3	48	66,7	0	0
**31**	Post-teat treatment with Chlorhexidin	16	22,2	56	77,8	0	0
**32**	Post-teat treatment with another medium	24	33,3	48	66,7	0	0
**33**	Dip	39	54,2	33	45,8	0	0
**34**	Spray	29	40,3	43	59,7	0	0
**35**	Milking cluster disinfection after each cow	42	58,3	30	41,7	0	0
**36**	Seperate milking parlor for sick cows	4	5,6	68	94,4	0	0
**37**	Waste milk cows milked at the end	46	63,9	25	34,7	1	1,4
**38**	Benestermycin	45	62,5	27	37,5	0	0
**39**	Orbenin	13	18,1	59	81,9	0	0
**40**	Other antibiotic dry therapeutica	14	19,4	58	80,6	0	0
**41**	Use of teat sealants	61	84,7	11	15,3	0	0
**42**	Dry period ≥ 42 days	64	88,9	8	11,1	0	0
**43**	Dry period < 42 days	7	9,7	65	90,3	0	0
**44**	Maternal vaccinations	46	63,9	26	36,1	0	0
**44a**	Rota/Corona/*E. coli*	44	61,1	28	38,9	0	0
**44b**	Clostridia	5	6,9	67	93,1	0	0
**45**	Isolated calving on deep bedding	12	16,7	60	83,3	0	0
**46**	Co-calving on deep bedding	67	93,1	5	6,9	0	0

**47**	Co-calving in cubicle system housing	2	2,8	70	97,2	0	0
**48**	Co-calving on sand bedding	1	1,4	71	98,6	0	0
**49**	Cleaning after each birth	2	2,8	70	97,2	0	0
**50**	Cleaning > 1 x per week	23	31,9	49	68,1	0	0
**51**	Cleaning < 1 x per week	45	62,5	27	37,5	0	0
**52**	Calves < 1 h with dams	24	33,3	48	66,7	0	0
**53**	Calves 1-3 h with dams	37	51,4	35	48,6	0	0
**54**	Calves > 3 h with dams	11	15,3	61	84,7	0	0
**55a**	Co-husbandry of calving cows	59	81,9	13	18,1	0	0
**55b**	Co-husbandry of calving and sick cows	12	16,7	60	83,3	0	0
**56**	Single calf husbandry	61	84,7	11	15,3	0	0
**57**	Co-husbandry	4	5,6	68	94,4	0	0
**58**	Single and co-husbandry	7	9,7	65	90,3	0	0
**59**	Igloo-husbandry	44	61,1	28	38,9	0	0
**60**	Pen-husbandry	21	29,2	51	70,8	0	0
**61**	Mixed Igloo and pen husbandry	4	5,6	68	94,4	0	0
**62**	No change in husbandry	22	30,6	50	69,4	0	0
**63**	Change after 7 days	11	15,3	61	84,7	0	0
**64**	Change after 14 days	30	41,7	42	58,3	0	0
**65**	Change after 21 days	3	4,2	69	95,8	0	0
**66**	Concrete floor	69	95,8	3	4,2	0	0
**67**	Daily bedded with straw	21	29,2	51	70,8	0	0
**68**	Direct contact to other calves	62	86,1	10	13,9	0	0
**69**	Calve-feedlot Ratio >1:1	2	2,8	70	97,2	0	0
**70**	Further animal contact	70	97,2	2	2,8	0	0
**71**	All-in-all-out-principle only ♀	2	2,8	70	97,2	0	0
**72**	Colostrum immediately after birth	14	19,4	58	80,6	0	0
**72a**	Within 3 h after birth	45	62,5	27	37,5	0	0
**72b**	More than 3 h after birth	13	18,1	59	81,9	0	0
**72c**	Sucked from the own dam	2	2,8	70	97,2	0	0
**72d**	Milked from the own dam	43	59,7	29	40,3	0	0
**72e**	Stored in refrigerator	20	27,8	52	72,2	0	0
**72f**	Stored in freezer	11	15,3	61	84,7	0	0
**72g**	Pasteurized	2	2,8	70	97,2	0	0
**72h**	Acidified	3	4,2	69	95,8	0	0
**72i**	First meal ≤ 2 L	7	9,7	65	90,3	0	0
**72j**	First meal >2 < 4 L	32	44,4	40	55,6	0	0
**72k**	First meal ≥ 4 L	33	45,8	39	54,2	0	0
**72l**	Drench	38	52,8	34	47,2	0	0
**72m**	Nursing bucket	29	40,3	43	59,7	0	0
**72n**	Mix of drench and nursing bucket	5	6,9	67	93,1	0	0
**773**	Quality check colostrum	38	52,8	34	47,2	0	0

**73a**	Refractometer	27	37,5	44	61,1	1	1,4
**73b**	Colostrometer	10	13,9	61	84,7	1	1,4
**74**	Cows > 3 weeks *a.p.*present in farm	69	95,8	3	4,2	0	0
**75**	Identical feeding ♂ ♀	58	80,6	14	19,4	0	0
**76**	Milk pasteurized	13	18,1	59	81,9	0	0
**77**	Milk acidified	15	20,8	57	79,2	0	0
**78**	Milk pasteurized and acidified	6	8,3	66	91,7	0	0
**79**	2 meals / day	52	72,2	20	27,8	0	0
**80**	3 meals / day	9	12,5	63	87,5	0	0
**81**	> 3 meals / day	11	15,3	61	84,7	0	0
**82**	< 5 L / day	44	61,1	28	38,9	0	0
**83**	> 5 L / day	2	2,8	70	97,2	0	0
**84**	ad libitum feeding	26	36,1	46	63,9	0	0
**85**	Waste milk + antibiotic residues (WM)	12	16,7	60	83,3	0	0
**86**	Raw milk	8	11,1	64	88,9	0	0
**87**	Milk replacer (MR)	14	19,4	58	80,6	0	0
**88**	WM + MR	24	33,3	48	66,7	0	0
**89**	WM + Raw milk	7	9,7	65	90,3	0	0
**90**	WM + Raw milk + MR	5	6,9	67	93,1	0	0
**91**	Raw milk + MR	2	2,8	70	97,2	0	0
**92**	WM as part of the feeding	48	66,7	24	33,3	0	0
**93**	Fixed feeding time	64	88,9	8	11,1	0	0
**94**	Use of automatic feeder	8	11,1	64	88,9	0	0
**95**	Cleaning of the feeder	59	81,9	13	18,1	0	0
**95a**	With water	21	29,2	51	70,8	0	0
**95b**	With cleaning agent	19	26,4	53	73,6	0	0
**95c**	With disinfectant	4	5,6	68	94,4	0	0
**95d**	With cleaning agent + disinfectant	10	13,9	62	86,1	0	0
**95e**	After each meal	15	20,8	57	79,2	0	0
**95f**	1 x per day	21	29,2	51	70,8	0	0
**95g**	< 1 x per day	23	31,9	49	68,1	0	0
**96**	Supplementary feed	71	98,6	1	1,4	0	0
**96a**	Roughage before 14th day of life	28	38,9	44	61,1	0	0
**96b**	Concentrate before 14th day of life	43	59,7	29	40,3	0	0
**96c**	Roughage after 14th day of life	17	23,6	55	76,4	0	0
**96d**	Concentrate after 14th day of life	22	30,6	50	69,4	0	0
**96e**	Only female calves receive roughage and concentrate	1	1,4	71	98,6	0	0
**97**	Tap water for MR	23	31,9	49	68,1	0	0
**98**	Well water for MR	26	36,1	46	63,9	0	0
**99**	Free water access	61	84,7	11	15,3	0	0
**99a**	Tap water	27	37,5	45	62,5	0	0

**99b**	Well water	37	51,4	35	48,6	0	0
**100**	≤ 2 persons take care of the calves	36	50	36	50	0	0
**101**	Trained personnel	60	83,3	12	16,7	0	0
**102**	Untrained personnel	33	45,8	39	54,2	0	0
**103**	Constant personnel	65	90,3	4	5,6	3	4,2
**104**	Changing personnel	7	9,7	65	90,3	0	0
**105**	Protective clothing	15	20,8	57	79,2	0	0
**106**	Preventive treatment for calves	61	84,7	11	15,3	0	0
**107**	Treatment schedules for calves	44	61,1	27	37,5	1	1,4
**108**	Preventive treatment for cows	50	69,4	22	30,6	0	0
**109**	Treatment schedules for cows	65	90,3	7	9,7	0	0
**110**	Antibiotic treatment for every clinical mastitis	36	50	36	50	0	0
**111**	Antibiotic treatment <50% of clinical mastitis	5	6,9	67	93,1	0	0
**112**	Change of antibiotics only after completion of treatment	51	70,8	21	29,2	0	0
**113**	Change of antibiotics ≤ 3 d	21	29,2	51	70,8	0	0
**114**	Alternative treatment methods	21	29,2	51	70,8	0	0
**115**	Co-husbandry of waste milk cows and the dairy herd	16	22,2	56	77,8	0	0
**116**	Separate husbandry of waste milk cows	55	76,4	17	23,6	0	0
**117**	Use of cleaning agents	5	6,9	67	93,1	0	0
**118**	Dry-through	5	6,9	65	90,3	2	2,8
**119**	Use of disinfectant	10	13,9	62	86,1	0	0
**120**	Use of lime	18	25	54	75	0	0
**121**	Cleaning and disinfectant plan	6	8,3	66	91,7	0	0
**122**	Use of cleaning agents	38	52,8	34	47,2	0	0
**123**	Dry-through	57	79,2	13	18,1	2	2,8
**124**	Use of disinfectant	58	80,6	14	19,4	0	0
**125**	Use of lime	3	4,2	69	95,8	0	0
**126**	Cleaning and disinfectant plan	21	29,2	51	70,8	0	0
**127**	Use of cleaning agents	48	66,7	24	33,3	0	0
**128**	Dry-through	5	6,9	67	93,1	0	0
**129**	Use of disinfectant	10	13,9	62	86,1	0	0
**130**	Use of lime	2	2,8	70	97,2	0	0
**131**	Cleaning and disinfection plan	18	25	54	75	0	0
**132**	Daily antibiotic use on <2/100 animals	65	90,3	5	6,9	2	2,8
**133**	Daily antibiotic use on 2-5/100 animals	5	6,9	65	90,3	2	2,8
**134**	Use of beta-lactam antibiotics	72	100	0	0	0	0
**135**	Use of Tetracyclines	67	93,1	5	6,9	0	0
**136**	Use of Aminoglycosides	62	86,1	9	12,5	1	1,4

**137**	Use of Polypeptide antibiotics	0	0	72	100	0	0
**138**	Use of Macrolides	20	27,8	51	70,8	1	1,4
**139**	Use of Fenicols	29	40,3	42	58,3	1	1,4
**140**	Use of Folic acid antagonists	40	55,6	31	43,1	1	1,4
**141**	Use of Quinolones	40	55,6	31	43,1	1	1,4
